# Rehabilitation Succeeds Where Technology and Pharmacology Failed: Effective Treatment of Persistent Pain across the Lifespan

**DOI:** 10.3390/jcm8122042

**Published:** 2019-11-21

**Authors:** Kelly Ickmans, Lennard Voogt, Jo Nijs

**Affiliations:** 1 Pain in Motion International Research Group, Department of Physiotherapy, Human Physiology and Anatomy, Faculty of Physical Education & Physiotherapy, Vrije Universiteit Brussel, Laarbeeklaan 103, 1090 Brussels, Belgium; l.p.voogt@hr.nl (L.V.); jo.nijs@vub.be (J.N.); 2 Department of Physical Medicine and Physiotherapy, Universitair Ziekenhuis Brussel, Laarbeeklaan 101, 1090 Brussels, Belgium; 3 Research Foundation-Flanders (FWO), 1000 Brussels, Belgium; 4 Research Centre for Health Care Innovations, Rotterdam University of Applied Sciences, 3015 EK Rotterdam, The Netherlands; 5 Department of Physical Therapy Studies, Rotterdam University of Applied Sciences, 3015 EK Rotterdam, The Netherlands

Chronic pain affects up to 30% of the adult population [1] and 11% to 38% of the childhood and adolescent population [2,3]. Its tremendous personal and socioeconomic impact is reflected by its cause of the highest number of years lived with disability [4] and being the most expensive cause of work-related disability in adults [5,6]. In children and adolescents, chronic pain causes decreased participation in recreational activities, difficulty maintaining social contacts, school absence and academic impairment, decreased health related quality of life, and increased health care utilization [3,7].

The area of rehabilitation research for patients having persistent pain is on the move with a substantial increase in the scientific understanding of persistent pain over the past decades. This rapid growth in pain science has inspired rehabilitation clinicians and researchers around the globe, leading to breakthrough research and the implementation of contemporary pain science in rehabilitation settings. Still, our understanding of persistent pain continues to grow, not in the least because of fascinating discoveries from areas such as psychoneuroimmunology, epigenetics, exercise physiology, clinical psychology, and nutritional (neuro)biology. This offers unique opportunities to further improve rehabilitation for patients with chronic pain. As age is a determining factor in the uniqueness of the bio-, psycho-, and social factors of persistent pain, this also implies that rehabilitation interventions should be tailored across the lifespan. Also, the diversity of health care disciplines involved in the rehabilitation of chronic pain (e.g., physicians, psychologists, physiotherapists, occupational therapists, nurses, coaches) provides a framework for upgrading rehabilitation for chronic pain towards comprehensive lifestyle approaches.

A number of articles published in this Special Issue draw specific attention to interdisciplinary multimodal rehabilitation programs for chronic pain. Ringqvist et al. [8] provide evidence that such programs delivered to adults in specialist care show moderate long-term effect sizes for pain, pain interference in daily life, and perceived health. Interestingly, Pfeifer et al. [9] provide preliminary support for the utility of incorporating an attachment-informed approach within these existing multimodal pain therapies, thereby aiming at advancing the working alliance between patient and therapist. In the realm of pediatric chronic pain rehabilitation, Harrison et al. [10] state that preliminary evidence on interdisciplinary outpatient treatments is promising with regard to improvements in pain intensity, pain-related disability, school attendance, catastrophizing, and symptoms of depression. In addition, addressing multiple unfavorable lifestyle factors, such as physical inactivity, sedentary behavior, stress, smoking, unhealthy diet, and poor sleep concomitantly, seems to be a challenge for which such interdisciplinary pain rehabilitation programs may offer a comprehensive framework. Indeed, unfavorable lifestyle factors and pain have been shown to be interconnected [11]. This suggests that multimodal lifestyle-centered approaches may be effective for chronic pain. Actually, this matter is touched on in each of the five invited contributions on the best evidence rehabilitation for chronic pain [10,12,13,14,15], thus underscoring its topicality for persistent pain rehabilitation and providing important avenues for future research.

The invited contributions in this Special Issue are part of a “Best Evidence Rehabilitation for Chronic Pain” Series comprising five state-of-the-art papers from world leading experts regarding persistent pain. Part 1, by Harrison et al. [10], covers the current state-of-the-art rehabilitation approaches to treat persistent pain in children and adolescents. In addition, several emerging areas of interventions are highlighted to guide future research and clinical practice. Part 2, the article by De Groef et al. [12], provides the reader with a state-of-the-art overview of the best evidence rehabilitation modalities for patients having (persistent) pain during and following cancer treatment. This paper is of particular importance to the field of oncology, especially now that common practices to manage cancer pain are being challenged due to a lack of supporting evidence [16,17]. In parts 3 and 4, Malfliet et al. [13] and Sterling et al. [14] present an overview of the best evidence non-invasive rehabilitation for people having chronic low back pain and neck pain, respectively. Finally, in part 5, a state-of-the-art review of rehabilitation for osteoarthritis pain is provided by Rice et al. [15]. For each of these domains, the best evidence rehabilitation is reviewed in a way that clinicians can integrate it into their daily clinical routine. The “Best Evidence Tables”, “Future Directions for Clinical Practice” sections, and key references to treatment manuals included in each of these papers serve to meet that aim. In addition, these overview articles also help clinical researchers to build upon the best evidence for designing future trials, implementation studies, and new innovative studies.

In summary, the collection of high-quality work presented in this Special Issue provides important new evidence from experimental lab-based as well as clinical studies, all focusing on rehabilitation for people with persistent pain. The review articles included in the “Best Evidence Rehabilitation for Chronic Pain” Series together delineate an important trend of continuously growing evidence supporting rehabilitation approaches for people with chronic pain. The more rehabilitation programs for people with chronic pain develop into multimodal lifestyle approaches, the stronger the evidence supporting them as key elements in the treatment for chronic pain. This is in sharp contrast with medical interventions for chronic pain such as (spinal) surgery, interventional treatments such as radiofrequency denervation, and analgesics that struggle following rigorous scientific evaluation [18,19,20], especially when side effects and cost-effectiveness are taken into account. Rehabilitation is succeeding where technology and pharmacology failed: providing effective treatment for people suffering from chronic pain. Still, much work needs to be done regarding implementation as well as scientific research. Therefore, we believe that the original and novel information along with the overview papers within the “Best Evidence Rehabilitation for Chronic Pain” Series in this Special Issue will serve as an important resource for researchers and an aid for clinicians to facilitate integration from research into daily clinical practice. Thereby, we hope to serve as a guiding light for future research in this area and to aid in further improvements in the quality of care for people with persistent pain across the lifespan.
